# PreBINDS: An Interactive Web Tool to Create Appropriate Datasets for Predicting Compound–Protein Interactions

**DOI:** 10.3389/fmolb.2021.758480

**Published:** 2021-12-06

**Authors:** Kazuyoshi Ikeda, Takuo Doi, Masami Ikeda, Kentaro Tomii

**Affiliations:** ^1^ Medicinal Chemistry Applied AI Unit, HPC- and AI-driven Drug Development Platform Division, RIKEN Center for Computational Science, Yokohama, Japan; ^2^ Division of Physics for Life Functions, Keio University Faculty of Pharmacy, Tokyo, Japan; ^3^ Lifematics Inc., Tokyo, Japan; ^4^ Artificial Intelligence Research Center (AIRC), National Institute of Advanced Industrial Science and Technology (AIST), Tokyo, Japan; ^5^ AIST-Tokyo Tech Real World Big-Data Computation Open Innovation Laboratory (RWBC-OIL), National Institute of Advanced Industrial Science and Technology (AIST), Tokyo, Japan

**Keywords:** compound-protein interaction, CHEMBL, machine learning, interactive web server, deep learning, datasets

## Abstract

Given the abundant computational resources and the huge amount of data of compound–protein interactions (CPIs), constructing appropriate datasets for learning and evaluating prediction models for CPIs is not always easy. For this study, we have developed a web server to facilitate the development and evaluation of prediction models by providing an appropriate dataset according to the task. Our web server provides an environment and dataset that aid model developers and evaluators in obtaining a suitable dataset for both proteins and compounds, in addition to attributes necessary for deep learning. With the web server interface, users can customize the CPI dataset derived from ChEMBL by setting positive and negative thresholds to be adjusted according to the user’s definitions. We have also implemented a function for graphic display of the distribution of activity values in the dataset as a histogram to set appropriate thresholds for positive and negative examples. These functions enable effective development and evaluation of models. Furthermore, users can prepare their task-specific datasets by selecting a set of target proteins based on various criteria such as Pfam families, ChEMBL’s classification, and sequence similarities. The accuracy and efficiency of *in silico* screening and drug design using machine learning including deep learning can therefore be improved by facilitating access to an appropriate dataset prepared using our web server (https://binds.lifematics.work/).

## 1 Introduction

Identification of disease-causing proteins and compounds that act on those diseases is an important starting point in the drug discovery process (Hughes et al., 2011). Over the last two decades, the amounts of compound–protein interaction (CPI) data have been increasing rapidly because of advances in experimental techniques such as high-throughput screening (HTS) (Bleicher et al., 2003; Macarron et al., 2011). Considering the social effects of the spread of infectious diseases, as exemplified by COVID-19, early discovery of therapeutic agents is highly anticipated (Cui et al., 2019). Improving drug development efficiency using known CPI data is necessary because it can shorten times to market and reduce costs.

Machine learning (ML) methods using CPI data have already been regarded as effective means for the hit-to-lead stage (Ghasemi et al., 2018; Ferreira and Andricopulo, 2019). In recent years, the development of artificial intelligence (AI) using deep learning has been remarkable. In fact, AI prediction models have already been applied to various issues; further enhanced efficiency of drug discovery is expected (Tsubaki et al., 2019; Beker et al., 2020; Kojima et al., 2020). In the field of ML-based CPI prediction research, some widely used benchmark datasets and development methods have been proposed (He et al., 2017; Wu et al., 2018; Rifaioglu et al., 2020, 2021).

The accuracy of prediction models is generally known to depend heavily on the quality and quantity of training data. Nevertheless, it is often not easy to obtain a suitable dataset for creating a reliable prediction model. Particularly in the fields of biochemistry and medicinal chemistry, it is difficult for non-specialists to obtain high-quality CPI data and to distinguish between positive and negative cases.

A widely used database of bioactive molecules is ChEMBL. Because the collected data are derived mainly from the literature, they include various CPI data points with different activity types and values (Mendez et al., 2019). Although the ChEMBL interface provides a useful function for searching and extracting downloadable activity data, it is unsuitable for directly generating positive/negative datasets for ML. PubChem provides free access to obtain large amounts of CPI data from results of screening experiments (Kim et al., 2021). However, these data are not provided to users as a binary format that can be used easily for ML. The chemical structures obtained from these databases are provided by the Simplified Molecular Input Line Entry System (SMILES) (Weininger, 1988) and MDL molfile (Dalby et al., 1992). Also, LIT-PCBA (Tran-Nguyen et al., 2020) was released as a structure-based virtual screening benchmark dataset. It also provided for MOL2 and SMILES formats. Therefore, to use it as input for ML, one must use chemical calculation programs such as CDK (Willighagen et al., 2017)^1^. Then the data must be encoded into physicochemical properties and structural descriptors (fingerprints). As a result, users must bear heavy burdens to prepare suitable CPI datasets for their research.

For this study, we have developed a web server that simplifies creation of CPI datasets for the development and evaluation of prediction models. Because the compound data relies on curated ChEMBL data, it includes high-quality chemical data such as drug-like small molecule compounds. The target data are based on proteins from the UniProt database (Bateman et al., 2021), which is well known as a reviewed protein sequence database. We also provide classifications of target proteins based on the Pfam clan (Mistry et al., 2021), ChEMBL’s protein target classification, and sequence similarity. This web server provides a new environment that enables developers to build and evaluate their prediction models more effectively and which might reduce costs of finding drug candidates.

## 2 Materials and Methods

### 2.1 Preparation of Compound Attributes

We used ChEMBL to obtain chemical data of compounds. From ChEMBL release 28, bioactive compounds associated with single protein targets were collected in the molfile format as compound structural data. Next, using Open Babel (O’Boyle et al., 2011) implemented in RDKit (ver. 2020.09.3), a library for chemical calculations, the compound structure data was converted to four fingerprints: Extended Connectivity Fingerprints four and 6 (ECFP-4 and ECFP-6) (Rogers and Hahn, 2010), MACCS keys, and FP2.

### 2.2 Preparation of CPI Data and Protein Attributes

Based on the ChEMBL activity data, we extracted target proteins with protein IDs (i.e., UniProt accession numbers). We collected only activity data that were assigned pChEMBL values (negative logarithms of activity values in nM units). Next, sequence data were obtained from the UniProt Knowledgebase. Protein families and domains for the target proteins were annotated with the cross-reference information from the Pfam database (release 33.1). Each target sequence was labeled with its accession number. Because the attribute (feature) of a target protein, its Position-Specific Scoring Matrix (PSSM) was calculated using the Blast+ 2.11.0 program with adjustment (Oda et al., 2017) search against the UniRef90 database (Suzek et al., 2007), which provides hiding of redundant sets of sequences from UniProt.

### 2.3 Clustering Methods

To provide a diverse set of compounds, we clustered the compounds using the MiniBatch–KMeans method of the scikit-learn program (0.22.1) based on ECFP4 fingerprint similarity. Here, we set *K* = 10,000. Target proteins were clustered in terms of their sequence similarity. We clustered the collected target proteins using different similarity thresholds (40, 50, 90%) with Cluster database at high identity with tolerance (CD-HIT, v4.8.1; Li and Godzik, 2006), which is a fast and efficient program for clustering large-scale protein sequences. Next, we pre-computed and registered the clustering data into the internal database. As a result, users can easily and quickly select a set of representative target proteins having sequence identity higher than a specified similarity threshold on the interface.

### 2.4 Representative Negative Sample Generation

When selecting negative examples, we used a method that generates a set of negatives that do not resemble the known compounds of a given target. The ChEMBL data are generally known to include more positive data than negative data, which is unbalanced as a training dataset for activity prediction. Therefore, we modified a method for generating reliable negative data (Liu et al., 2015). A credible negative sample is based on the assumption that proteins differing from the known and predicted targets of a particular compound are unlikely to be the targets of that compound. A representative negative set was extracted from a cluster to which positive data do not belong. The negative prediction model was made using support vector machine (SVM). The scikit-learn package sklearn.svm.SVC was used for the SVM calculation.

### 2.5 Schema and Data Contents

The database schema comprises 11 tables (Supplementary Figure S1). There are five table groups: ChEMBL derived data (compounds, activities, assays), UniProt target annotation data, Pfam family data, clustering results of proteins, and ligand mapping data. As shown in Table 1, this web server contains 1.33 million compounds (chemical molecules) and 8,341 single protein targets. These protein targets were classified into 2,733 Pfam families, and not all but many of them belong to 635 Pfam clans. UniChem (Chambers et al., 2013) was used to identify the ligands found in the Protein Data Bank (Berman et al., 2000). Currently, 12,980 PDB ligands have been registered in this system.

**TABLE 1:** Data contents.

Type	Count
Compounds	1,331,700
Assays	338,454
Targets	8,341
Pfam families	2,733
Pfam clans	635
CD-HIT clusters (90%)	6,649
CD-HIT clusters (50%)	4,504
CD-HIT clusters (40%)	3,890
PDB ligands	12,980

## 3 Results and Discussion

The web server was built as a relational database management system using Python 3.8.10 programming language, Django 3.2.2, Vue 2.6.11, and SQLite. It provides a simple and interactive graphical user interface (GUI).

### 3.1 Web Interface

**FIGURE 1:**
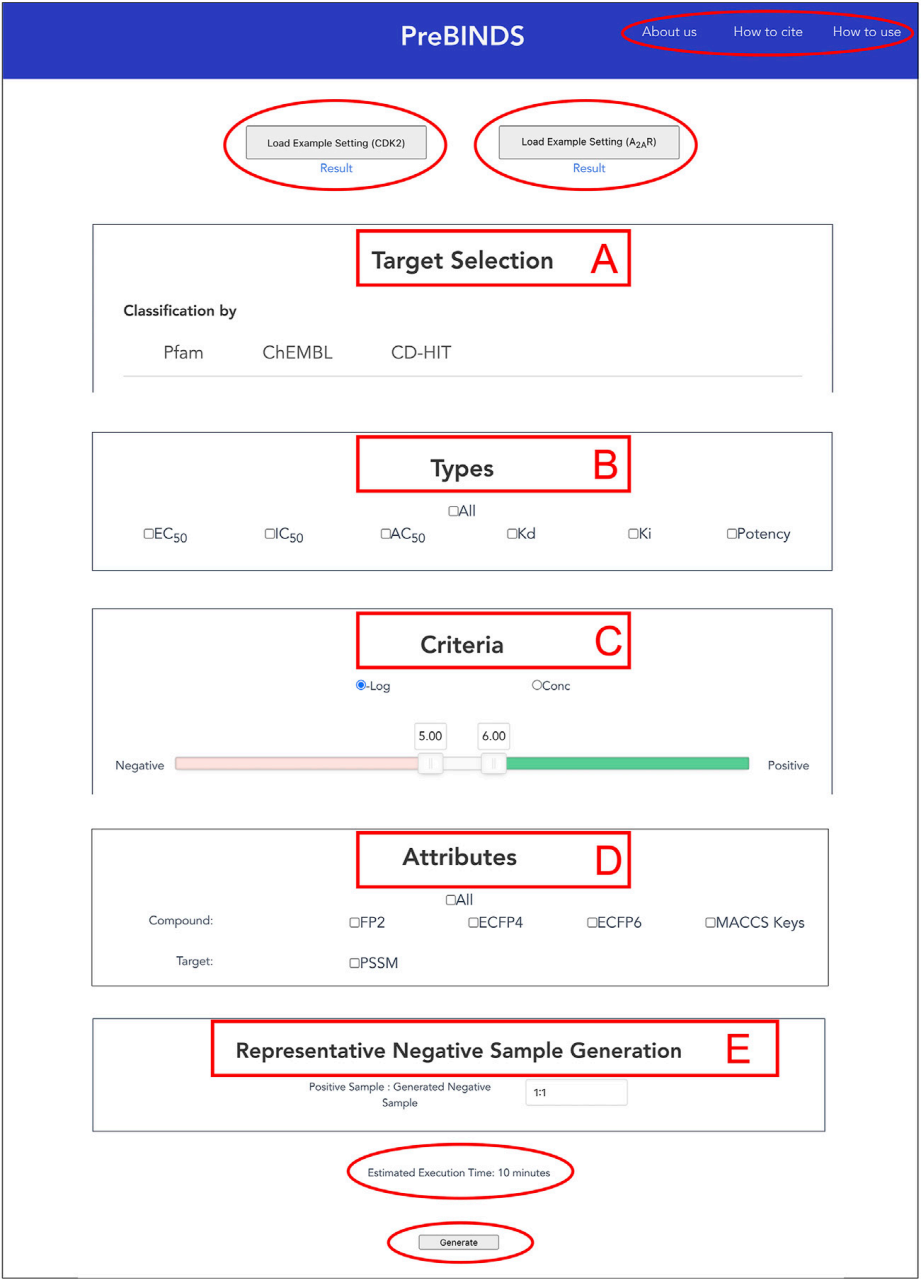
Partial view of the web server interface, which is designed to be simple and easy to use, allowing users to select and retrieve compound–protein interaction datasets quickly and interactively. Users configures their dataset in five steps: **(A)** Select protein targets (“Target Selection”), **(B)** Select activity types (“Types”), **(C)** Set activity data thresholds (“Criteria”), **(D)** Select compound and target attributes for output (“Attributes”), and **(E)** Set and generate negative data (“Representative Negative Sample Generation”). At the top of the interface, the “How to use” link leads to a brief instruction of this web server. “About us” and “Cite us” links respectively lead to information of developers and citations of this web server. “Load Example Setting” buttons provide sample setting parameters for generating datasets of two representative targets (A2AR and CDK2) and download links to obtain the output files. At the bottom of the interface, users can find the estimated time to complete the submitted dataset generation job in advance.

Users can use the interface easily by displaying the top page without logging in (Figure 1). The interface allows users to search for protein targets and to create datasets for obtaining data. The procedure on this web tool includes the following steps: 1) selection of target proteins (“Target Selection”), 2) selection of activity types (“Types”), 3) setting of criteria for activity values (“Criteria”), 4) selection of attributes (“Attributes”), 5) generation of negative data samples, and 6) download of output data. The example data button (load example setting) has been implemented. In the load example setting function, the target is set to A_2A_ receptor or CDK2. The appropriate target proteins, activity types, activity values and margin, attributes, and negative sample setting are given. With this feature, users can test the web server. We also implemented a download button to provide output data. With this feature, users can readily understand the results from the web server in advance.

#### 3.1.1 Selection of Target Proteins

**FIGURE 2:**
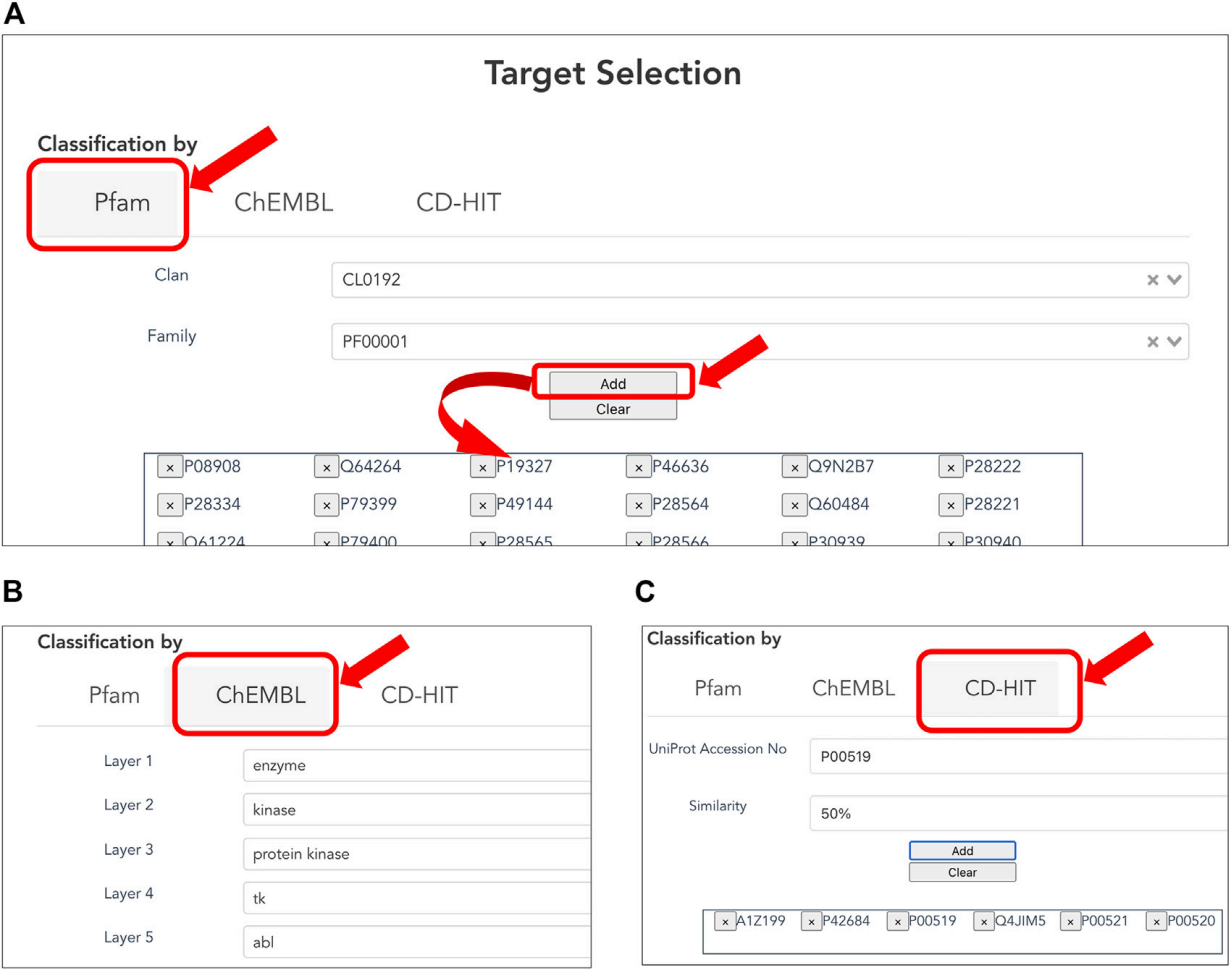
Selecting target proteins. **(A)** Selection of protein families according to Pfam clans. A list of protein families is displayed by the selected Pfam clan. Users can select one of the Pfam IDs and press the “ADD” button to provide the corresponding protein accessions to the form at the bottom. **(B)** Selection of target proteins according to ChEMBL target classification. **(C)** Selection of target proteins by sequence similarity based on pre-computed CD-HIT clustering results. The user can select the sequence similarity threshold for clustering from 90, 50, and 40% to provide the corresponding protein accessions.

Users can customize the list of target proteins to generate a CPI dataset (Figures 2A–C) based on classification of three types below. The Pfam family is classified based on the similarity of domains, which are functional regions in proteins. The Pfam clan is a group of Pfam families that have been inferred as evolutionarily related. The collected targets were annotated using domain and clan/family data extracted from the Pfam database (ver. 33.1). When creating a CPI dataset in terms of the evolutionary relevance of target proteins, users can select the target protein(s) of interest in the list of Pfam families according to Pfam’s classification (Figure 2A). When creating a CPI dataset in terms of biological function and pharmacology, users can select the target protein(s) of interest according to the ChEMBL target classification. The classification relies on the ChEMBL’s protein target classification, which is a hierarchical classification and manually defined by experts in the field of drug discovery based on protein functions, enzymatic reactions, pharmacological actions, and so on (Gaulton et al., 2012). In addition, users can use pre-computed CD-HIT clustering results to select target proteins by sequence similarity and generation of a list of target proteins (Figure 2C). The resulting target proteins are listed as UniProt accessions. We have also implemented the ability to delete each UniProt accession. With this deletion capability, a user can easily customize the list of UniProt accessions for the target proteins.

#### 3.1.2 Selection of Activity Types

**FIGURE 3:**
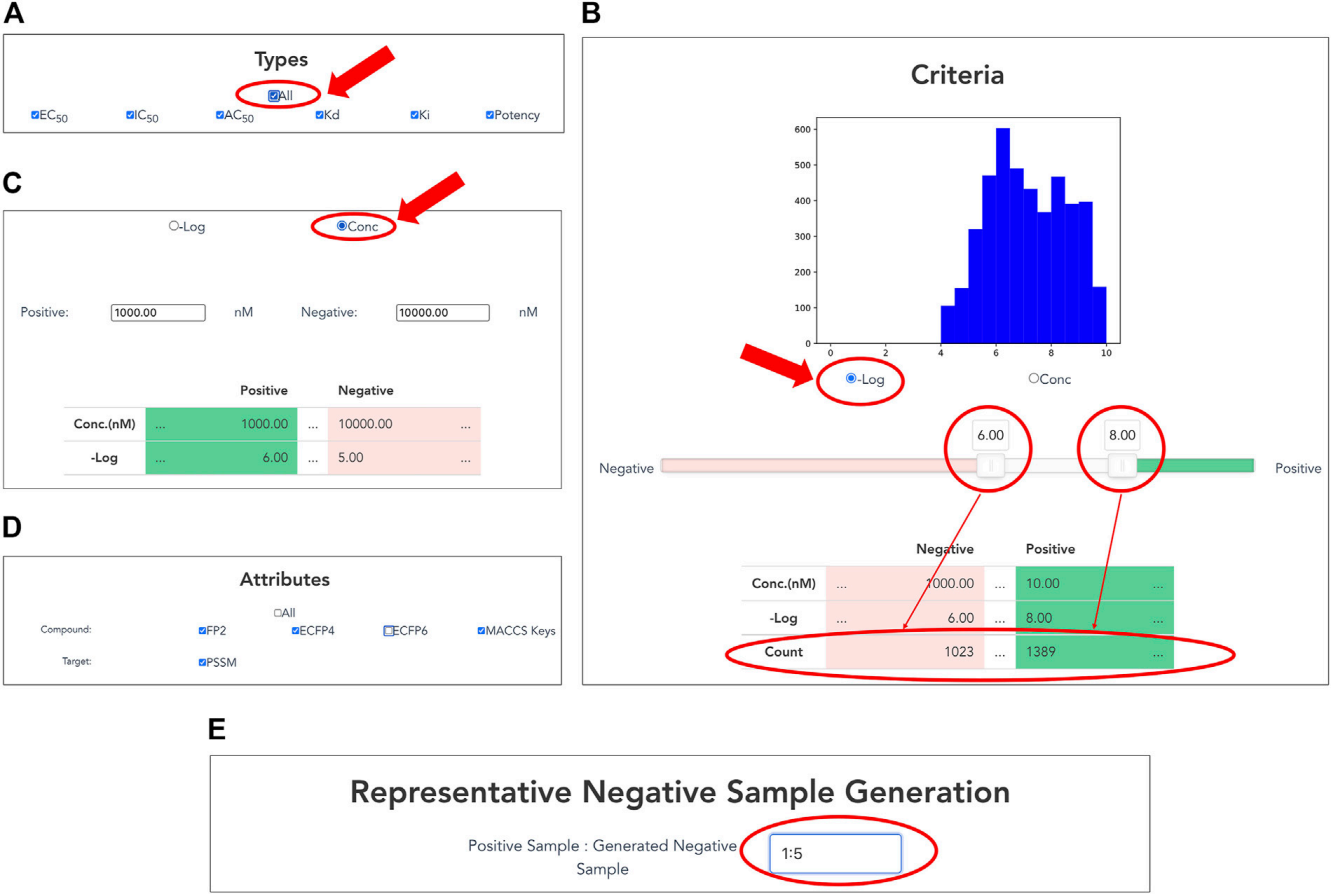
Customizing compound–protein interaction datasets. Activity data are filtered by activity types and a range of activity values. **(A)** Selection of activity types from EC_50_, IC_50_, AC_50_, Kd, Ki, and potency. **(B)** Setting thresholds for negative logarithms of activity values in nM units. Users can easily set thresholds for positive and negative data using a slide bar if selecting “−Log” of radio button. The graphical display of the histogram of the activity values and the counter of the number of activity values help the user to set the appropriate threshold values. **(C)** Users can directly input concentration thresholds for activity values (nM) of positive and negative data if checking “Conc” of radio button. **(D)** Selection of compound and protein attributes for output. Users can select compound attributes from ECFP4, ECFP6, FP2, and MACCS keys (Canonical SMILES by default), as well as protein attributes as PSSM (protein sequence by default). **(E)** Generation of representative negative samples. Users can customize the amount of negative data for output. This function allows users to select the amount of negative sample generated from 1, 3, and 5 times the amount of the positive sample.

Users can customize datasets by specifying activity types and the range of activity values on the interface (Figures 3A–C). Users select a single or multiple activity type(s) among EC_50_, IC_50_, AC_50_, Kd, Ki, and potency for the activity data associated with the selected targets (Figure 3A). In addition, the "All” button allows the user to select all options easily.

#### 3.1.3 Setting of Criteria for Activity Values

By inputting the upper and lower thresholds of the activity value, positive and negative data are definable. On the interface, the activity value is displayed as the negative logarithm (-Log) (Figure 3B) or the concentration of dose–response experiment (nM) (Figure 3C). Users can use a slider bar to set their thresholds easily for positive and negative data. In addition, the range of the margin between positive and negative datasets can be set arbitrarily by assignment of upper and lower thresholds of concentration. A counter has also been incorporated, showing the exact number of bioactive data points in the positive and negative datasets. This feature makes it easy for the user to visualize using the histogram with the number of active values for the training set. It is expected to be particularly helpful when adapting to ML. For many targets, setting a low threshold of activity value increases the number of compounds for learning, but it might include non-specific binders. It is generally known that proper thresholds and margins differ depending on targets. Therefore, this function helps moderate a dataset for effective prediction models.

#### 3.1.4 Selection of Attributes for Output

Users choose attributes of proteins and compounds in the CPI data for output (Figure 3D). As a compound attribute, users can select from a single or multiple molecular fingerprint(s) (ECFP4, ECFP6, FP2, and MACCS key). The canonical SMILES of the selected compounds were outputted without selecting any option. As a target attribute, protein sequence is output by default; PSSM can be additionally selected.

#### 3.1.5 Representative Negative Sample Generation

Users can customize the amount of negative data to output. This function allows the users to choose between 1, 3, and 5 times the amount of generated negative samples compared to number of positive samples. Details of the procedure are presented in the *Materials and Methods* section.

#### 3.1.6 Download of Output Data

**FIGURE 4:**
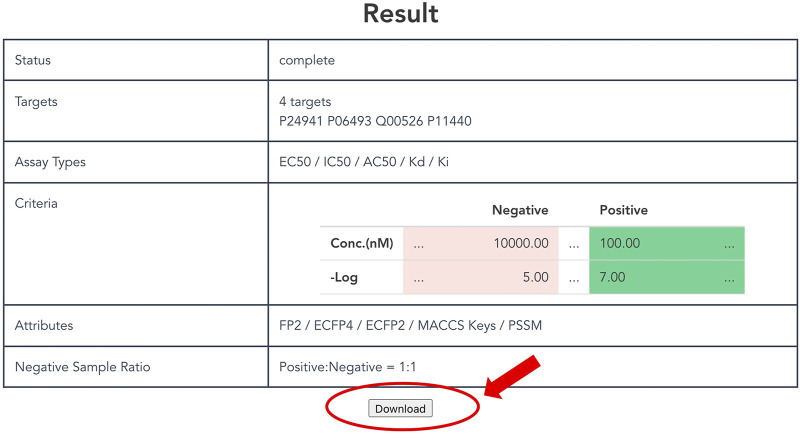
Display of result page. The result page shows the progress of the submitted dataset generation job and the input parameters specified by the user. A “Download” button appears at the bottom of this page when the job is completed, allowing the user to download the dataset.

As described above, a non-redundant CPI dataset can be prepared according to attributes and thresholds. Users click the “Generate” button at the bottom of the top page to output a customized dataset. It might take some time to request generation of the dataset, so users can confirm the selected items and the status on the result page (Figure 4). Finally, after clicking the download button, users can obtain a compressed file formatted dataset that includes protein and compound attributes.

## 4 Conclusion

A web server has been developed for generating datasets of compound–protein interactions. This web server can provide a ready-to-use CPI dataset for ML, including deep learning in drug discovery and development. Obtaining the latest compound and activity data from the ChEMBL and other public databases is important for developing accurate prediction models. For this reason, our web server shall be updated regularly. Additional information will be imported promptly from external resources. The web server is expected to be useful for developing and evaluating ML models for predicting protein–compound interactions, and for discovery of new bioactive molecules.

## Data Availability

Publicly available datasets were analyzed in this study. This data can be found here: https://binds.lifematics.work/.

## Footnotes

1
http://www.rdkit.org[2020.09.3]

## Supplementary Material

Supplementary Figure S1 Database schema of this web server.
