# Supplementary figures and images for: PreBINDS: An Interactive Web Tool to Create Appropriate Datasets for Predicting Compound–Protein Interactions
Source: Front Mol Biosci. 2021 Dec 6;8:758480. doi: 10.3389/fmolb.2021.758480 (PMC8685504; doi:10.3389/fmolb.2021.758480)

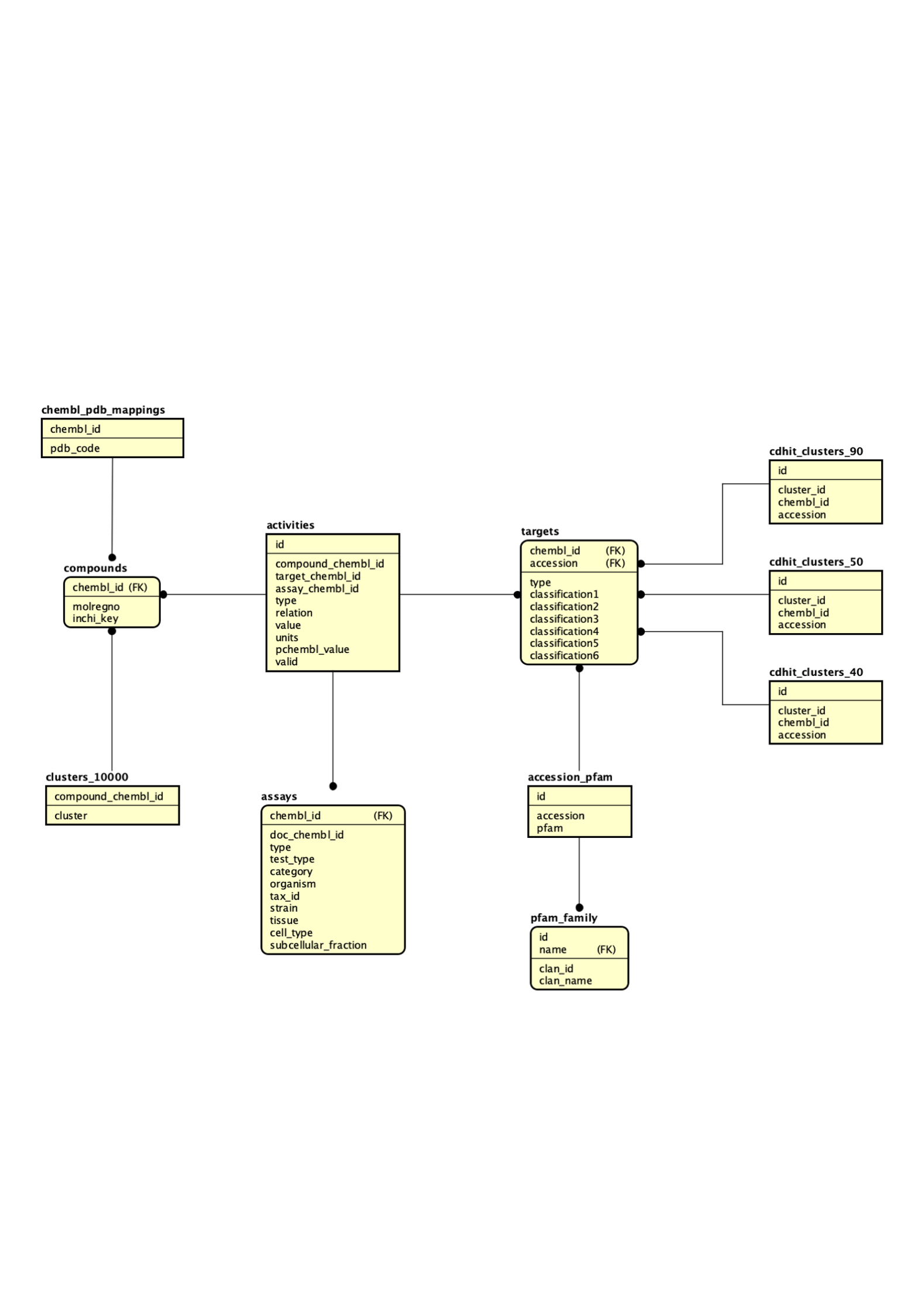

Supplement: Supplementary file 1 [file Image1.TIF]
